# Otic Neurogenesis Is Regulated by TGFβ in a Senescence-Independent Manner

**DOI:** 10.3389/fncel.2020.00217

**Published:** 2020-08-17

**Authors:** Marta Magariños, Raquel Barajas-Azpeleta, Isabel Varela-Nieto, Maria R. Aburto

**Affiliations:** ^1^Institute for Biomedical Research “Alberto Sols” (IIBM), Spanish National Research Council-Autonomous University of Madrid (CSIC-UAM), Madrid, Spain; ^2^Centre for Biomedical Network Research (CIBER) on Rare Diseases (CIBERER), Institute of Health Carlos III, Madrid, Spain; ^3^Department of Biology, Universidad Autónoma de Madrid, Madrid, Spain; ^4^Hospital La Paz Institute for Health Research (IdiPAZ), Madrid, Spain; ^5^APC Microbiome Ireland, University College Cork, Cork, Ireland

**Keywords:** senescence, TGFβ2, inner ear development, inner ear neurogenesis, organotypic culture

## Abstract

Cellular senescence has classically been associated with aging. Intriguingly, recent studies have also unraveled key roles for senescence in embryonic development, regeneration, and reprogramming. Developmental senescence has been reported during embryonic development in different organisms and structures, such as the endolymphatic duct during inner ear development of mammals and birds. However, there is no study addressing the possible role of senescence on otic neurogenesis. TGFβ/SMAD is the best-known pathway linked to the induction of developmentally programmed cell senescence. Here, we studied if TGFβ2 induces cellular senescence during acoustic-vestibular-ganglion (AVG) formation. Using organotypic cultures of AVG, and characterizing different stages of otic neurogenesis in the presence of TGFβ2 and a selective TGF-β receptor type-I inhibitor, we show that TGFβ2 exerts a powerful action in inner ear neurogenesis but, contrary to what we recently observed during endolymphatic duct development, these actions are independent of cellular senescence. We show that TGFβ2 reduces proliferation, and induces differentiation and neuritogenesis of neuroblasts, without altering cell death. Our studies highlight the roles of TGFβ2 and cellular senescence in the precise regulation of cell fate within the developing inner ear and its different cell types, being their mechanisms of action highly cell-type dependent.

## Introduction

The vertebrate inner ear is a complex sensory organ responsible for the perceptions of sound and balance. The transmission of these auditory and balance information to higher brain regions relies on the auditory and the vestibular ganglia. These otic neurons extend their processes connecting the sensory epithelium to the brainstem nuclei through the eighth cranial nerve (Fekete and Campero, 2007; Fritzsch et al., 2015). During development, otic neuroblasts firstly gather together as the acoustic-vestibular ganglion (AVG), by delaminating from the neuro-sensory domain in the epithelium of the otocyst (Camarero et al., 2003; Magariños et al., 2012). In the context of embryonic development, the process of cellular senescence, although classically linked with aging and cancer (Collado et al., 2007), has been recently described to have functional roles from various animal species. Cellular senescence is a form of irreversible cell cycle arrest combined with phenotypic changes and pronounced secretory activity (Gorgoulis et al., 2019). Senescent cells express inhibitors of cyclin-dependent kinases, as p21, p15, and p16, whereas the expression of cell cycle-promoting genes is suppressed. Senescent cells also show increased expression of the lysosomal β-galactosidase enzyme, a feature manifested as stronger senescence-associated β-galactosidase staining (SAβG), the most widely used marker (Hernandez-Segura et al., 2018), and present a secretory profile known as the senescence-associated secretory phenotype (SASP) that include soluble factors that modulate the cellular microenvironment (He and Sharpless, 2017). Developmentally programmed senescence acts together with apoptosis in the elimination of unwanted cells as well as in patterning and morphogenesis (Lorda-Diez et al., 2015). It shares most of the cellular characteristics of replicative and oncogene-induced senescence, such as arrested proliferation, increased SAβG staining, and a secretory phenotype. However, so far only p21 appears to be critical for developmental senescence (Gorgoulis et al., 2019). Although initially developmental senescent cells were reported to lack DNA-damage markers (Muñoz-Espín et al., 2013), it was later reported that the interdigital limb tissue from chicken embryos is associated with phosphorylated -H2AX (Montero et al., 2016). Current data suggest that senescent cells may have multiple functions in the embryo (Da Silva-Álvarez et al., 2019; Rhinn et al., 2019). These development-associated senescent cells arise in very precise patterns in time and space, before subsequently disappearing, pointing to a tightly regulated appearance and removal of these cells (Muñoz-Espín et al., 2013; Storer et al., 2013; Davaapil et al., 2017). Some of the structures that have been associated with programmed cellular senescence are the endolymphatic sac, the mesonephros, the neural tube, the apical ectodermal ridge, the interdigital tissue and the endolymphatic duct (Muñoz-Espín et al., 2013; Storer et al., 2013; Lorda-Diez et al., 2015; Gibaja et al., 2019; Rhinn et al., 2019).

This cellular process constitutes a part of a set of highly organized events that drive embryogenesis. Mechanistically, cellular senescence has been linked to the TGFβ/SMAD and PI3K/FOXO pathways. Particularly, the transforming growth factor-beta (TGFβ) signaling pathway has been described to be key in regulating programmed senescence in the context of the degenerating mesonephros (Muñoz-Espín et al., 2013) and inner ear morphogenesis (Gibaja et al., 2019; Varela-Nieto et al., 2019).

The TGFβ superfamily of growth factors is vital for the development and homeostasis of metazoans (Feng and Derynck, 2005). Members of this superfamily are extremely well conserved throughout evolution and they regulate diverse cellular functions such as migration, programmed cell death, differentiation, growth, and adhesion. Moreover, these actions are extremely well-orchestrated in time and space during development (Wu and Hill, 2009). The general signal transduction pathway for these growth factors consists basically of ligand dimers binding and activating heteromeric complexes of type I and type II transmembrane receptors, which in turn phosphorylate the intracellular mediators (SMADs). These mediators form complexes between each other and with other proteins, to regulate target gene expression (Wharton and Derynck, 2009; Wu and Hill, 2009). TGFβ superfamily has been also described to play central roles in multiple aspects of nervous system development and function. Among the various members of this superfamily, TGFβ2 has been involved in regulating the temporal identity and potency of neural stem cells (Dias et al., 2014), in retinal neuronal survival (Walshe et al., 2011; Braunger et al., 2013), proliferation of granule cells in the cerebellum, neuronal migration of glioma cells and axon elongation in hippocampal neurons, among others (Meyers and Kessler, 2017). Other members of the TGFβ superfamily have been widely studied in the otic vesicle (Kamaid et al., 2010; Ohyama et al., 2010). In this study, we aimed to explore whether TGFβ2 also plays a role in inner ear neurogenesis and if so, whether these effects are also mediated, at least partially, by cellular senescence. Our results show that TGFβ2 exerts a powerful action in the inner ear neurogenesis, but these actions are independent of cellular senescence, contrary to what we recently observed during endolymphatic duct development. We show that TGFβ2 reduces proliferation, and induces differentiation and neuritogenesis of neuroblasts, without altering cell death.

## Materials and Methods

### Chicken Embryos

Chicken embryos were obtained from fertilized eggs from a local farm (Granja Santa Isabel, Cordoba, Spain) and they were incubated in a humidified atmosphere at 37.8°C. Embryos were staged as HH15, HH18, and HH19 according to Hamburger and Hamilton’s criteria (Hamburger and Hamilton, 1951).

### Embryo and Tissue Preparation for *in situ* Hybridization and Immunofluorescence

Whole embryos or tissues were dissected in phosphate-buffered saline (PBS) and fixed overnight in 4% (w/v) paraformaldehyde (PFA; Merck, Darmstadt, Germany) in PBS at 4°C. Subsequently, embryos were cryoprotected overnight in 15% sucrose/PBS at 4°C and then embedded at 37°C in 15% sucrose/10% gelatine in PBS. Gelatine-embedded tissues were frozen in isopentane at −80°C and then sectioned (20 μm) at −25°C in a cryostat (Cryocut 1900; Leica Microsystems, Deerfield, IL, USA). The sections were obtained from 3–6 embryos and used for *in situ* hybridization or immunofluorescent staining as described Aburto et al., 2012b).

### Isolation, Organotypic Culture, and Treatment of Otic Vesicles and Acoustic-Vestibular Ganglia

Otic vesicles were obtained from HH18 embryos (65 h of incubation), by dissecting them from the surrounding mesenchymal tissue with sharpened tungsten needles. Subsequently, they were transferred into four-well culture-plates (Delta treated for adhesion culture conditions and non-treated for regular culture assays; Nunc Roskilde, Denmark) and then incubated at 37°C in a water-saturated atmosphere containing 5% CO_2_ for the times indicated in the text. The standard culture medium consisted of M199 medium with Earle’s salts (Sigma-Aldrich, Saint Louis, MO, USA) supplemented with 2 mM glutamine (Gibco, Paisley, UK) and antibiotics [50 IU/ml penicillin (Ern, Barcelona, Spain) and 50 mg/ml streptomycin (CEPA, Madrid, Spain)]. For immunostaining and TUNEL labeling, otic vesicles were fixed for 2 h in PFA at 4°C. For SAβG staining the otic vesicles were fixed for 10 min with the fixative solution provided by the Senescence β-Galactosidase Staining Kit (Cell Signaling Technology, Danvers, MA, USA).

AVG was dissected from HH19 chicken embryos (82 h of incubation) and plated onto glass coverslips that had been previously coated with poly-D-lysine and fibronectin (Davies, 2011). AVG was cultured in 0.25 ml F12/Dulbecco’s modified Eagle medium (Gibco) containing 100 μg/ml transferrin, 16 μg/ml putrescine, 6 ng/ml progesterone, 5.2 ng/ml sodium selenite (all from Sigma) and antibiotics as described (Aburto et al., 2012a,b). Explants were treated with TGFβ2 diluted in medium (10 ng/ml, PeproTech, Rocky Hill, NJ, USA). AVG explants cultured in medium and adding the equivalent volume of medium without TGFβ2 was used as experimental controls (0S). Three to six AVG explants per condition were assayed.

### Immunohistochemistry and Immunofluorescence

Details of the antibodies used for immunofluorescence are shown in Supplementary Table S1. Procedures used have been described elsewhere (Magariños et al., 2010; Aburto et al., 2012a,b). Briefly, samples were washed and permeabilized in 1% PBS/Triton-X-100 (PBS-T) non-specific binding sites were blocked for 1 h in PBS-T with 3% (wt/vol) BSA (Sigma-Aldrich, Saint Louis, MO, USA) and 5% (vol/vol) normal goat or donkey serum. Samples were incubated with the primary antibodies overnight at 4°C, diluted in PBS-Tween20 (0.05%). For immunofluorescent staining of frozen sections, the primary antibodies were used as described above and the secondary antibodies [Alexa Fluor 488 goat anti-mouse (1:200), Alexa Fluor 647 goat anti-rabbit and/or Alexa Fluor 546 goat anti-rabbit secondary antibodies (1:200; all from Molecular Probes, Eugene, OR, USA)] were incubated at room temperature for 1.5 h (frozen sections) or 2.5 h (otic vesicle and AVG explants). The samples were mounted in Prolong Gold with DAPI (Invitrogen, Carlsbad, CA, USA) and visualized by fluorescence (Nikon 90i, Tokyo, Japan) or confocal microscopy (Leica TCS SP2, Wetzlar, Germany).

Levels of SOX2, number of PHH3+ cells, G4 glycoprotein intensity levels and AVG areas were quantified using ImageJ software (Wayne Rasband, National Institutes of Health, Bethesda) in compiled confocal microscopy projections as reported (Aburto et al., 2012a,b; Magariños et al., 2010). The color channels of the signals of interest were converted into grayscale images. Subsequently, both the area and the intensity of the signal were measured and normalized to the 0S condition, which was given an arbitrary value of 100. Three to six samples per condition were assayed. The data are presented as the mean ± SEM and the statistical significance was estimated with Student’s *t*-test.

### TUNEL Assay

Apoptotic cell death patterns were studied by Tdt-mediated dUTP nick-end labeling (TUNEL) of fragmented DNA using the kit Dead-EndTM Fluorometric TUNEL System (Promega, Madison, WI, USA), as described by the manufacturer in frozen sections as reported (Frago et al., 2003), or adapted to whole organ labeling (Camarero et al., 2003; Frago et al., 2003). Otic vesicles were mounted in Prolong Gold/DAPI and visualized by confocal microscopy. TUNEL-positive nuclei were quantified from compiled confocal microscopy projections by FIJI software, and the results are presented as the mean ± SEM of the positive cells per total area. Values were normalized to those of the 0S explants. Three to five AVG per condition were assayed.

### *In situ* Hybridization

*In situ* hybridization with digoxigenin-labeled antisense RNA probes (1 mg/ml) was performed essentially as described previously with some minor modifications (Sánchez-Calderón et al., 2004). Three embryos of the stages HH15 and HH18 were tested in parallel in at least two independent experiments. The probes utilized in this study are *TGFB* and *TGFBR2*. The specificity of the signal was assessed by hybridization of a sense riboprobe.

### Senescence-Associated Beta-Galactosidase Staining

The senescence-associated beta-galactosidase staining (SAβG) was performed by using the Senescence β-Galactosidase Staining Kit (Cell Signaling Technology, Danvers, MA, USA). After culture and fixation, the otic vesicles were washed in PBS and incubated in the X-Gal solution at pH 6 at 37°C protected from light. For SAβG in whole embryos at stages HH18 and HH19, the embryos were fixed for 15 min and incubated in the X-Gal solution. To perform the SAβG in chicken embryo tissue sections, the embryos were obtained, fixed for 15 min, frozen, and sectioned with a cryostat by the Histology Core of the “Centro Nacional de Biotecnología.” Sections were post-fixed for 2 min and incubated with the X-Gal solution.

For SAβG staining of otic vesicles, explanted otic vesicles were fixed with the fixative solution provided in the SAβG kit for 8 min and incubated with the X-Gal solution at pH 6 at 37°C. Then, otic vesicles were fixed with 4% PFA, permeabilized with PBS-T, washed with 2% PBS-BSA and incubated with the Click-iT Reaction Cocktail (Invitrogen) with Alexa-488 azide (5 μM, Invitrogen) at RT in darkness for 30 min. Finally, they were washed again with 2% PBS-BSA and mounted in Vectashield with DAPI (Vector, Peterborough, UK).

### Image Analysis and Statistical Analysis

At least three to six otic vesicles or AVG per condition were used for each experiment, dissected from at least three different chicken embryos. Fluorescence intensities and areas of interest were quantified using ImageJ software (Wayne Rasband, National Institutes of Health, Bethesda) in compiled confocal microscopy projections as reported (Aburto et al., 2012a,b). Subsequently, both the intensity of the signal and the area were normalized to the 0S condition, which was given an arbitrary value of 100. The data are presented as the mean intensity, or the mean number of positive cells per total area, ± SEM. The statistical significance was estimated using Student’s *t*-test.

## Results

### Expression of the TGFβ Pathway Elements During Inner Ear Neurogenesis

**Figure 1:**
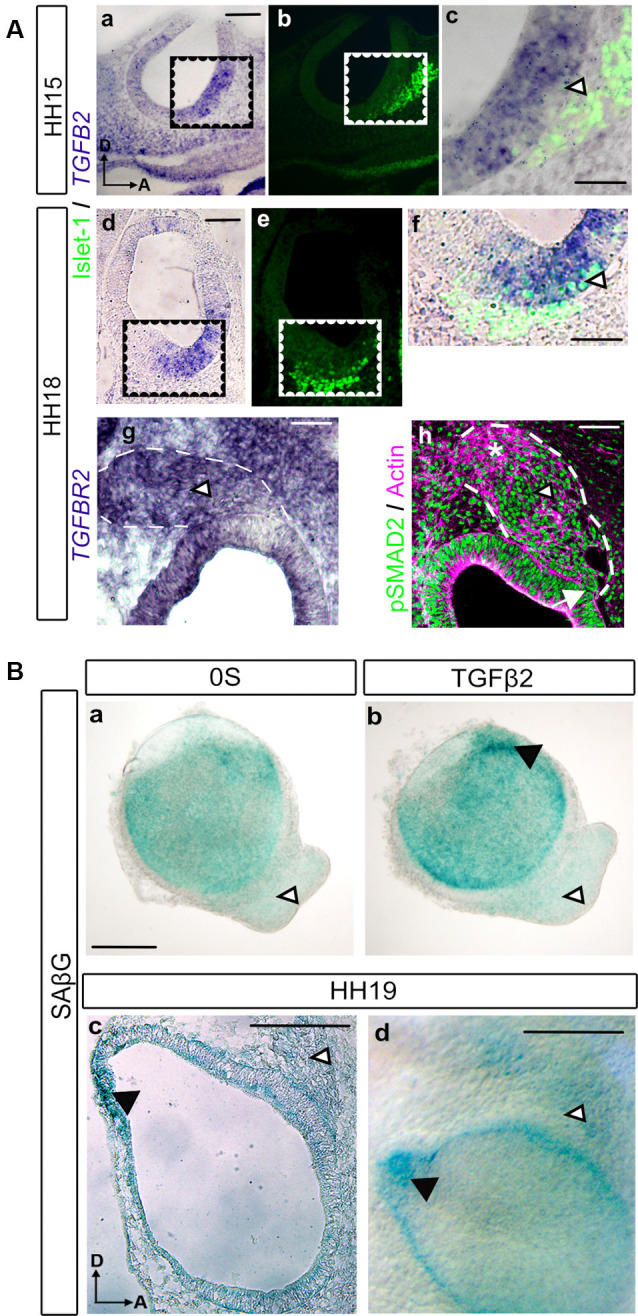
TGFβ pathway expression during early otic neurogenesis. **(A)** TGFβ pathway components (*TGFB2*, *TGFBR2* and pSMAD2) and neural markers expression in the developing inner ear. *TGFB2* *in situ* hybridization in transverse sections of chicken embryos. At HH15, *TGFB2* was expressed in the otic neurogenic region (**a**, cropped area in **c**). The associated neuroblasts are labeled with Islet-1 (green in **b,c**). At HH18 *TGFB2* continues to be present in the otic neurogenic region (**d**, cropped area in **f**) associated to the neuroblasts marker Islet-1 (green in **e,f**). Also, at HH18, *TGFBR2* showed a marked presence throughout the acoustic-vestibular-ganglion (AVG; **g**, arrowhead). Immunostaining for activated SMAD (pSMAD) showed nuclear immunopositivity in the otic epithelium and in the acoustic-vestibular ganglion (green in **h**, arrow and arrowhead, respectively). Actin was used as a marker of general structure in the OV and AVG area (magenta in **h**). Scale bars 75 μm **(a,b,d,e)** or 30 μm **(c,f,g,h)**. **(B)** Otic vesicles were isolated from HH18 chicken embryos and cultured for 20 h in **(a)** serum-free medium (0S) or **(b)** in the presence of TGFβ2 (10 ng/ml). SAβG staining was used to detect cellular senescence. TGFβ2 increases SAβG in the endolymphatic duct primordium (black arrowhead) while no SAβG+ cells were observed in the AVG with or without TGFβ2 treatment (white arrowheads); SAβG staining in a cryosection **(c)** or whole mount **(d)** HH19 chicken embryo is associated to the endolymphatic duct (white arrowheads) whereas no staining is detected in the AVG (black arrowheads). Scale bars 75 μm. Representative microphotographs are shown from at least three embryos and 3–6 isolated otic vesicles per condition. Orientation: A, anterior; D, dorsal.

TGFβ factors and their receptors are expressed in the developing inner ear (Gibaja et al., 2019). Here, we studied the expression pattern of the *TGFB2* transcript by *in situ* hybridization during the initial phases of inner ear development (Figures 1Aa–f). *TGFB2* mRNA presented a localized expression at both HH15 and HH18 stages in the otic epithelium, where the otic neuroblasts are being differentiated, known as the neurogenic region. This expression pattern partially co-localized with the neural marker Islet-1 (Figures 1Aa–f, arrowheads). We also explored the expression of the receptor *TGFBR2* at HH18, which showed a marked presence throughout the AVG (Figure 1Ag, arrowhead). This suggests that ganglionic neuroblasts can respond to TGFβ. Moreover, consistent with the activation of the TGFβ2 pathway, we found the typical nuclear immunopositivity for phosphorylated SMAD2 (pSMAD2) effector protein, both in the neurogenic region at the otic epithelium and in the AVG different ganglionic populations: the delaminating neuroblasts (Figure 1Ah, arrow), the trans-amplifying neuroblasts (Figure 1Ah, arrowhead) and the postmitotic neurons (Figure 1Ah, asterisk) of the AVG.

### TGFβ Pathway Does Not Induce Senescence in the AVG

As we previously described, TGFβ2 is a potent regulator of cellular senescence during inner ear development, which has been proven to be an essential process for proper morphogenesis of the endolymphatic duct (Gibaja et al., 2019). We, therefore, aimed to investigate whether developing AVG neuroblasts in HH19 stages underwent cellular senescence regulated by TGFβ2. β-galactosidase staining (SAβG) was used to detect senescent cells in the developing AVG, both in whole-mount embryos and cultured otic vesicles (Figure 1B). Whilst SAβG stained senescent cells at different areas of the otic epithelium, such as the endolymphatic duct Anlagen (Figure 1Bb, black arrowhead), we could not observe any senescent cells at the AVG under any of the culture conditions tested (Figures 1Ba,b, white arrowheads): control condition (0S) or in the presence of TGFβ2.

We also investigated the presence of senescent cells in the developing inner ear of HH19 embryos. A positive SAβG signal was evident in the early developing endolymphatic duct (Figures 1Bc,d, black arrowheads) as reported. In contrast, we could not find a clear SAβG signal at the AVG (Figures 1Bc,d, white arrowheads), following the *ex vivo* observations.

### TFGβ Pathway Is Required for the Differentiation of Acoustic Vestibular Neurons

**Figure 2:**
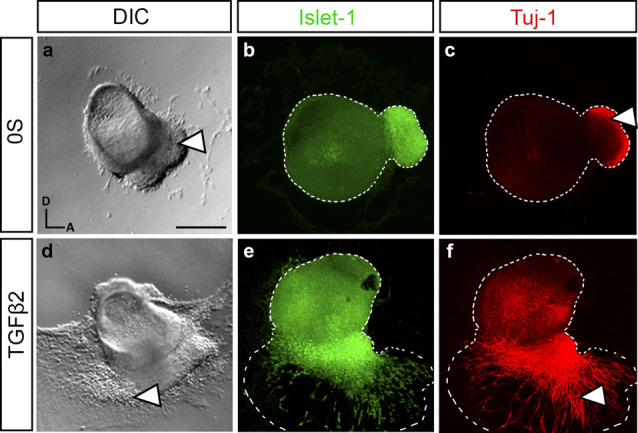
TGFβ2 modulates neurogenesis in cultured otic vesicles. Otic vesicles were isolated from HH18 chicken embryos and cultured for 20 h in serum-free culture medium without additions (0S, **a–c**) or in the presence of TGFβ2 (10 ng/ml, **d–f**). Double immunostaining was carried out for the neuroblast nuclear marker Islet-1 (green) and the post-mitotic marker Tuj-1 (red). Representative images of compiled confocal microscopy projections are shown from at least six otic vesicles per condition. Orientation: A, anterior; D, dorsal. Scale bar: 150 μm.

TGFβ2-treated otocysts in culture exhibited profound alterations in the newly-formed AVG. More precisely, we could observe a stronger attachment and increased migration of the ganglionic cells (Figures 2a,d, arrowheads). This was further studied by examining the expression of the neural markers Islet-1 and Tuj-1 in cultured explants treated with or without TGFβ2 (Figures 2b,c,e,f). Islet-1 is a transcription factor expressed by proliferative neuroblasts and showed increased expression both in the otic epithelium and AVG. Tuj-1 is a β-III-tubulin constituent of neural fibers of postmitotic neurons. With these markers, we confirmed that the TGFβ2-targeted cells were indeed AVG neuroblasts, which showed a greater spreading from the otocyst. Moreover, TGFβ2-treated AVG showed an increased and extended distribution of Tuj-1 (Figures 2c,f, arrowheads), suggesting an accelerated differentiation of proliferating Islet-1 positive neuroblasts to neurons.

**Figure 3:**
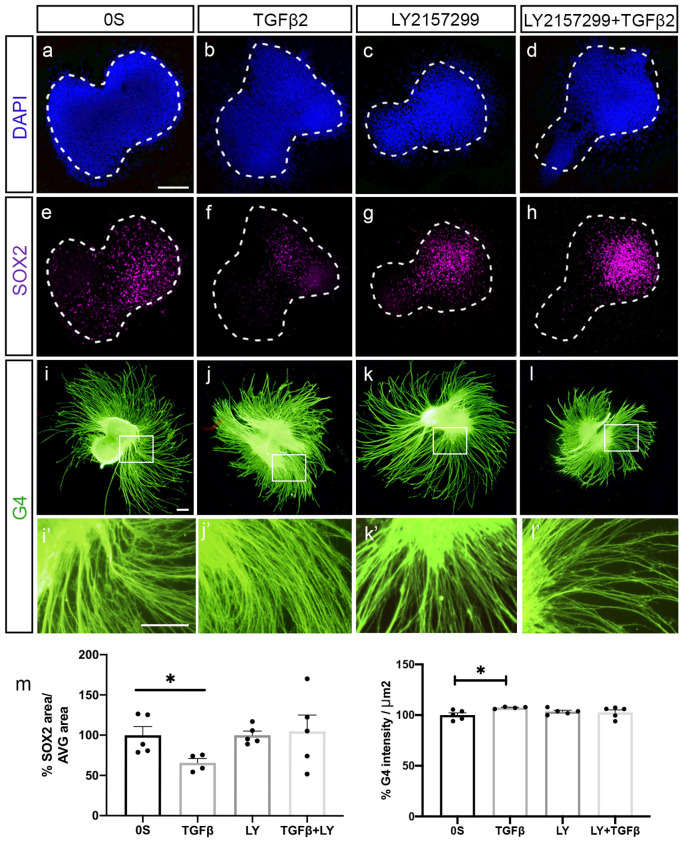
TGFβ2 promotes neural differentiation. **(a–l)** Acoustic-vestibular ganglia (AVG) cultured in serum-free medium (0S), in the presence of TGFβ2 (10 ng/ml), LY (10 μM) or a combination of both. Whole AVG explants were immunostained for the transcription factor SOX2 (magenta) expressed in neuroblasts and for the G4-glycoprotein used as a marker of neuronal processes (G4, green). Representative images of compiled confocal microscopy projections are shown of four to five AVG per condition. Scale bar 100 μm. **(m)** G4-mean intensity and SOX2-area were normalized and quantified as described in “Material and Methods” section, and the results are shown as the mean ± SEM relative to the 0S condition. Statistical significance was estimated with the Student’s *t*-test: *P < 0.05 vs. 0S.

To further verify if AVG neurite outgrowth was promoted by TGFβ2, we prepared cultures of explanted AVG (HH19) to study the differentiation of neuroblasts (Figure 3). Exogenous TGFβ2 enhanced the differentiation and neuritogenesis in ganglionic neuroblasts, as shown by the significant 7% increased levels of the neuron-specific glycoprotein G4 (Figures 3i,i′,j,j′ quantifications in **m**) and the 33% decreased levels of the transcription factor SOX2 (Figures 3e,f; quantifications in **m**). These differentiating effects of exogenous TGFβ2 were abrogated by a selective TGF-β receptor type I inhibitor, LY2157299 (Figures 3h,l; quantifications in **m**). The presence of the inhibitor alone did not show any effect compared to the control condition (Figures 3g,k).

**Figure 4:**
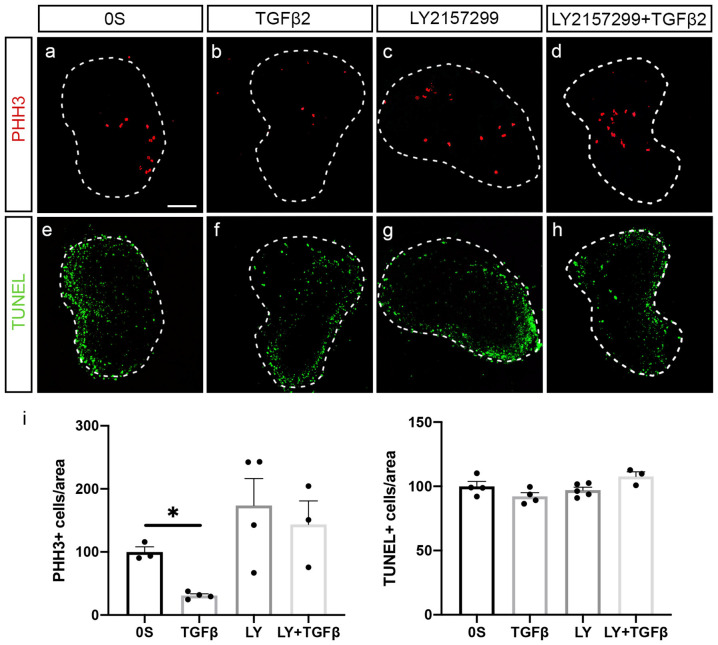
TGFβ2 decreases neural proliferation. **(a–h)** Acoustic-vestibular ganglia cultured in serum-free medium (0S), in the presence of TGFβ2 (10 ng/ml), LY (10 μM), or a combination of both. Apoptotic cell death was visualized by TUNEL (green), and proliferation was detected by immunofluorescence for the mitosis marker phospho-Histone 3 (PHH3, red). Representative images of compiled confocal microscopy projections are shown of three to five AVG per condition. Scale bar: 100 μm. **(i)** TUNEL-positive or proliferative-PHH3-labeled cells were quantified as described in “Materials and Methods” section, and the results are shown as the mean ± SEM relative to the 0S condition. Statistical significance was estimated with the Student’s *t*-test: *P < 0.05 vs. 0S.

TGFβ2 treatment showed a 69% significant reduction of proliferating neuroblasts measured by the mitotic marker phospho-Histone H3, PHH3 (Figures 4a,b, quantification in i). Furthermore, TGFβ2 inhibition by LY2157299 recovered the number of proliferating neuroblasts (Figures 4c,d, quantification in **i**). There was no evident alteration in the number of apoptotic cells (Figures 4e–h, quantification in **i**), suggesting that the observed decrease in proliferating neuroblasts was not due to increased cell death but to accelerated differentiation.

## Discussion

Cells with features of senescence have been identified in several transient anatomical structures in the developing embryo, and appear to play a role in shaping organogenesis (Muñoz-Espín et al., 2013; Storer et al., 2013; Davaapil et al., 2017; Gibaja et al., 2019; Rhinn et al., 2019; Varela-Nieto et al., 2019). However, to date, there is no report linking cellular senescence with neurogenic processes during embryogenesis. TGFβ2 has been previously described to promote the formation of the AVG (Okano et al., 2005), but a more insightful characterization of these effects was lacking. Thus, we aimed to gain a deeper understanding of the actions mediated by TGFβ2, by using organotypic cultures of AVG and characterizing the different stages of otic neurogenesis in the presence or absence of TGFβ2. We recently described an important role for TGFβ2 in regulating cellular senescence during the formation of the endolymphatic duct in vertebrates (Gibaja et al., 2019). We wondered if TGFβ2-mediated actions during the formation of the AVG could be also mediated, at least partially, by TGFβ2-induced cellular senescence.

In the present study, we offer insight into the key function of TGFβ2 in inner ear neurogenesis during embryonic development. We show that TGFβ2 regulates the differentiation of otic neuroblasts at the AVG by promoting cell cycle arrest, upregulation of neuronal markers such as G4 glycoprotein, and downregulation of the high mobility group (HMG) transcription factor SOX2. SOX2 is required for the initial events of otic neuronal specification, including the expression of the proneural basic helix-loop-helix transcription factor Neurogenin1 (Steevens et al., 2019). Thus, TGFβ2 signaling is necessary for the progression of otic neuroblasts differentiation into mature otic neurons. Otic neuroblasts derive from the neurosensory domain in the anteroventral region of the otic epithelium at the otic cup and otic vesicle stages (Magariños et al., 2012; Gálvez et al., 2017), which express SOX2. Interestingly, we show *TGFB2* mRNA expression precisely in the neurosensory domain, in which the otic neuroblasts are being specified and subsequently delaminate to give rise to the ganglionic neuroblasts. These results point to a role of TGFβ2 in promoting the progression of differentiation in neuroblasts. TGFβ2 also significantly reduced the number of proliferating neuroblasts in cultured AVG, further supporting the pro-differentiating actions of TGFβ2 on otic neuroblasts. According to our data, these actions seem to be independent of programmed cellular senescence, as we could not detect SAβG staining in the AVG from chicken embryos nor cultured otic vesicles. However, it has been also reported that it is possible to have senescent cells that do not stain with SAβG, as demonstrated in cells lacking *Glb1* (Rhinn et al., 2019). Importantly, some studies showed developmentally programmed senescence acting together with apoptosis (Lorda-Diez et al., 2015). Nonetheless, our results show that TGFβ2 does not alter cell death, supporting the idea that this molecule acts independently of programmed cellular senescence in the AVG. Nonetheless, to rule out the mechanistic action of TGFβ2 through cellular senescence in this context, deeper molecular studies, such as p21 expression or the secretory profile (SASP) of otic neuroblasts are needed. On the other hand, we could also detect lower levels of *TGFBR2* in the neurosensory domain, but we cannot discard the possibility that TGFβ2 actions in the neuroblast population are mediated through other receptors of the TGFβ superfamily, and/or that low levels of *TGFBR2* are sufficient to mediate these actions. Accordingly, we detected phosphorylated SMAD2 throughout the otic epithelium, as well as in the developing AVG. The TGFβR2 signal was very intense in the AVG, which may point towards the ability of the ganglionic neuroblasts to activate TGFβ signaling cascade. Alternatively, TGFβ2 secreted in the neuroepithelium could be acting directly on the ganglionic neuroblast population through TGFβ receptors, promoting the differentiation from trans-amplifying neuroblasts to post-mitotic neurons in the AVG.

Although the effects mediated by TGFβ2 in cultured otic vesicles or AVGs were both pointing to an enhanced maturation of otic neuroblasts, it is worth discussing the differences among the two systems. In otic vesicles, in the presence of TGFβ2, we could observe an enhanced adhesion of the organotypic culture to the substrate. Moreover, ganglionic neuroblasts presented a high migration rate away from the otic epithelium, as we could detect Islet-1-positive nuclei migrating forward the otic vesicle. These neuroblasts also expressed higher levels of Tuj-1. On the other hand, in the case of cultured AVG, there were no obvious effects on the migration rate of neuroblasts, but rather on their differentiation state. These differences can be possibly attributed to the different differentiation state of neuroblasts in the two culture systems tested, being otic vesicle neuroblasts in an earlier differentiation stage than those conforming to the AVG.

TGFβ-signaling has been also described to play key roles in regulating apoptosis during embryonic development. However, it is also clear that the death-inducing capacity of TGFβ is context-dependent, i.e., it is restricted to certain cell types, to a certain state of differentiation, and most notably, to the presence or absence of other growth factors (Schuster and Krieglstein, 2002). Examples of this, are the increased apoptosis in the endocardial cushions during the development of aorticopulmonary septum in mouse embryos upon elevated TGFβ2 levels (Kubalak et al., 2002) vs. the inhibition of apoptosis after TGFβ2 treatment in cultured retinal ganglion cells (Braunger et al., 2013). In our study, cultured AVG did not show alterations in the levels of apoptosis in the presence or absence of TGFβ2 nor in the presence of TGFβ-signaling inhibitor.

Understanding the molecular basis of developmental changes leading to the formation of the vertebrate auditory and vestibular ganglia is paramount to set a solid basis for efforts to regenerate this intricate organ in hearing loss patients (Chen et al., 2009, 2012). Our results add to this knowledge by showing that TGFβ2 promotes otic neuronal differentiation and that this does not involve the activation of cellular senescence. In contrast, previous results in our group showed that TGFβ2 does promote cellular senescence in the endolymphatic duct (Gibaja et al., 2019). Generally, our observations highlight the role of TGFβ2 in the precise regulation of cell fate within the developing inner ear and its different cell types. However, its mechanisms of action are highly cell-type dependent.

## Data Availability Statement

The data will be deposited in DIGITAL.CSIC (https://digital.csic.es), the institutional repository of the Spanish National Research Council.

## Ethics Statement

Ethical review and approval was not required for the animal study because DIRECTIVE 2010/63/EU does not consider foetal forms before last third of their development.

## Author Contributions

MA, IV-N, and MM designed the experiments and wrote the manuscript. MM, MA, and RB-A performed the experiments. MA, RB-A, IV-N, and MM analyzed and interpreted the data. All authors contributed to the article and approved the submitted version.

## Conflict of Interest

The authors declare that the research was conducted in the absence of any commercial or financial relationships that could be construed as a potential conflict of interest.
