# Supplementary material for: Otic Neurogenesis Is Regulated by TGFβ in a Senescence-Independent Manner
Source: Front Cell Neurosci. 2020 Aug 17;14:217. doi: 10.3389/fncel.2020.00217 (PMC7461926; doi:10.3389/fncel.2020.00217)
Supplement: Supplementary file 1 [file Table_1.DOCX]

**Table S1. List of antibodies and conditions used**

| ***Antibody*** | ***Type^1^*** | ***Source/Cat #*** | ***Concentration*** |
| --- | --- | --- | --- |
| **Anti-pSMAD2** | RbP | Cell Signaling/3101 | 1:200 (IHF) |
| **Anti-phospho-Histone 3** | Rbp | Upstate/06-570 | 1:200 (IHF) |
| **Anti-G4-Glicoprotein** | RbP | Ref.60-61 | 1:300 (IHF) |
| **Anti-SOX2** | GP | Santa Cruz/sc-17320 | 1:50 (IHF) |
| **Anti-Islet1** | MsM | DSHB/40.2D6 | 1:50 (IHF) |
| **Anti-TuJ1 (β-III Tubulin)** | RbP | Covance / PRB-435P | 1:500 (IHF) |

Summary of antibodies used for immunohistofluorescence and Western blotting. ^1^Antibody type: RbP, rabbit polyclonal; RbM, rabbit monoclonal; MouM, mouse monoclonal; GP, goat polyclonal. Abbreviations: IHF, immunohistofluorescence.
